# Chronological Age Interacts with the Circadian Melatonin Receptor 1B Gene Variation, Determining Fasting Glucose Concentrations in Mediterranean Populations. Additional Analyses on Type-2 Diabetes Risk

**DOI:** 10.3390/nu12113323

**Published:** 2020-10-29

**Authors:** Jose V. Sorlí, Rocío Barragán, Oscar Coltell, Olga Portolés, Eva C. Pascual, Carolina Ortega-Azorín, José I. González, Ramon Estruch, Carmen Saiz, Alejandro Pérez-Fidalgo, Jose M. Ordovas, Dolores Corella

**Affiliations:** 1 Department of Preventive Medicine and Public Health, School of Medicine, University of Valencia, 46010 Valencia, Spain; jose.sorli@uv.es (J.V.S.); rocio.barragan@uv.es (R.B.); olga.portoles@uv.es (O.P.); pascaseva89@gmail.com (E.C.P.); carolina.ortega@uv.es (C.O.-A.); ignacio.glez-arraez@uv.es (J.I.G.); carmen.saiz@uv.es (C.S.); j.alejandro.perez@uv.es (A.P.-F.); 2 CIBER Fisiopatología de la Obesidad y Nutrición, Instituto de Salud Carlos III, 28029 Madrid, Spain; oscar.coltell@uji.es (O.C.); restruch@clinic.cat (R.E.); 3 Department of Medicine, Sleep Center of Excellence, Columbia University Irving Medical Center, New York, NY 10032, USA; 4 Department of Computer Languages and Systems, Universitat Jaume I, 12071 Castellón, Spain; 5 Department of Internal Medicine, Hospital Clinic, Institut d’Investigació Biomèdica August Pi i Sunyer (IDIBAPS), University of Barcelona, Villarroel, 170, 08036 Barcelona, Spain; 6 CIBER Cáncer, Instituto de Salud Carlos III, 28029 Madrid, Spain; 7 Nutrition and Genomics Laboratory, JM-USDA Human Nutrition Research Center on Aging at Tufts University, Boston, MA 02111, USA; jose.ordovas@tufts.edu; 8 Precision Nutrition and Obesity Program, IMDEA Alimentación, 28049 Madrid, Spain

**Keywords:** melatonin receptor, fasting glucose, type-2 diabetes, MTNR1B polymorphism, age-interaction, heterogeneity, Mediterranean population, pregnancy, gestational diabetes, women

## Abstract

Gene-age interactions have not been systematically investigated on metabolic phenotypes and this modulation will be key for a better understanding of the temporal regulation in nutrigenomics. Taking into account that aging is typically associated with both impairment of the circadian system and a decrease in melatonin secretion, we focused on the melatonin receptor 1B (MTNR1B)-rs10830963 C>G variant that has been associated with fasting glucose concentrations, gestational diabetes, and type-2 diabetes. Therefore, our main aim was to investigate whether the association between the MTNR1B-rs10830963 polymorphism and fasting glucose is age dependent. Our secondary aims were to analyze the polymorphism association with type-2 diabetes and explore the gene-pregnancies interactions on the later type-2 diabetes risk. Three Mediterranean cohorts (*n* = 2823) were analyzed. First, a cross-sectional study in the discovery cohort consisting of 1378 participants (aged 18 to 80 years; mean age 41 years) from the general population was carried out. To validate and extend the results, two replication cohorts consisting of elderly individuals were studied. In the discovery cohort, we observed a strong gene-age interaction (*p* = 0.001), determining fasting glucose in such a way that the increasing effect of the risk G-allele was much greater in young (*p* = 5.9 × 10^−10^) than in elderly participants (*p* = 0.805). Consistently, the association of the MTNR1B-rs10830963 polymorphism with fasting glucose concentrations in the two replication cohorts (mean age over 65 years) did not reach statistical significance (*p* > 0.05 for both). However, in the elderly cohorts, significant associations between the polymorphism and type-2 diabetes at baseline were found. Moreover, in one of the cohorts, we obtained a statistically significant interaction between the MTNR1B polymorphism and the number of pregnancies, retrospectively assessed, on the type-2 diabetes risk. In conclusion, the association of the MTNR1B-rs10830963 polymorphism with fasting glucose is age-dependent, having a greater effect in younger people. However, in elderly subjects, associations of the polymorphism with type-2 diabetes were observed and our exploratory analysis suggested a modulatory effect of the number of past pregnancies on the future type-2 diabetes genetic risk.

## 1. Introduction

Currently, there is renewed interest in research into precision nutrition [1,2], an example being the initiative carried out by the National Institutes of Health (NIH) within the 2020–2030 Strategic Plan [3]. This Plan stresses the need for research into “when”. Research into “when” does not only consider the time of day [4] but also the age of the individuals [3]. This aspect is important, given that most studies that analyze the gen-diet interactions determining specific phenotypes do not usually take into account the possible heterogeneity relating to age [5,6,7]. In general, it is assumed that the effect of genetic polymorphisms, mainly single nucleotide polymorphisms (SNPs), is the same regardless of the age group analyzed. This can give rise to various biases and a lack of replication of associations on comparing some studies with others when they have been carried out using very different age groups. Furthermore, if later on, the gene-diet interactions of those polymorphisms are analyzed without taking into account the fact that their main effects on the initial phenotype may differ depending on the age of the population, this may lead to very uncertain conclusions.

Hence, an aspect that has to be analyzed in more detail before tackling a gene-diet interaction study is whether the main effects of a genetic polymorphism are homogeneous per age or if there is heterogeneity. Despite knowing that this is fundamental for its application in precision nutrition [1,2,3,8], few studies have tackled gene-age interactions [9,10,11,12,13,14]. Some of these studies have reported statistically significant gene-age interactions for several SNPs [9,10,11,12] on plasma lipids, blood pressure, and other related-phenotypes, highlighting the context-dependence of genetic effects, and demonstrate that displaying age-dependent effects can enhance our understanding of the temporal regulation of the main-related genes.

This paper focuses on the study of gene-age interactions of a very important gene related to chronobiology [15,16,17,18], the melatonin (5-methoxy-N-acetyltryptamine) receptor 1B (MTNR1B) gene, specifically the MTNR1B-rs10830963C>G polymorphism [19]. The variant allele G has been associated with higher fasting plasma glucose, gestational diabetes mellitus, and type-2 diabetes risk in various studies, with different results [20,21,22,23,24,25,26,27,28,29,30,31,32,33,34,35,36,37,38,39,40,41]. Although several polymorphisms in the MTNR1B gene, located on chromosome 11q21-q22, have been described [21], the MTNR1B-rs10830963 C>G polymorphism, in the intron between exon 1 and exon 2, is considered the most important. This variant is relatively frequent and some functionality has been reported (compared with the C allele, the variant G allele was associated with increased MTNR1B transcript levels and gene expression in human islets) [42,43], which likely leads to a reduction in insulin release, increasing type-2 diabetes risk [43]. However, the mechanisms involved are far from being elucidated. The melatonin best-known function is that of regulating circadian rhythms [44,45,46]. Desynchronization of the circadian rhythms has been related to an increased risk of diabetes [47,48,49]. Likewise, the MTNR1B-rs10830963 polymorphism is strongly associated with the development of gestational diabetes mellitus [34,35,36,37,38,40]. Gestational diabetes mellitus is a state of impaired glucose tolerance during pregnancy and is associated with future type-2 diabetes risk. Although it is known that pregnancy affects sleep quality and may disturb circadian rhythms [36,37,38], information about the interactive effects of the number of pregnancies on the genetic risk conferred by the MTNR1B-rs10830963 polymorphism on the future type-2 diabetes incidence is very scarce.

Circadian rhythms and sleep quality are closely regulated by melatonin, a crucial hormone determining the sleep-wake cycle in humans [18]. However, currently, there is considerable controversy over the favorable or unfavorable effects of melatonin on glucose metabolism and the risk of diabetes [50,51,52,53,54,55,56,57,58], considering that the results of studies both on humans and animals have often been inconsistent. In a recent review [58], these discrepancies were analyzed in-depth and one of the factors contributing to those discrepancies was the characteristics of the population studied, including chronological age [58].

After examining the chronological age of participants included in the corresponding published studies, we observed that the associations between the MTNR1B-rs10830963 polymorphism and fasting glucose concentrations were higher in investigations carried out in young populations [19,23,25,26]. In general, it is accepted that melatonin levels and melatonin receptor expression show a decrease during normal ageing and this reduction may be accelerated in some disease states [59,60]. Likewise, aging is associated with an impairment of the circadian system [18]. For all these reasons, we hypothesize that the age of the population studied may be an important factor when analyzing the effects of the MTNR1B-rs10830963 polymorphism on fasting glucose concentrations. Therefore, our first main aim was: (1) To investigate the interaction of the MTNR1B polymorphism with age in determining plasma glucose concentrations in a general population aged between 18 and 80 years (discovery cohort). Moreover, we had two additional secondary aims as follows: (2) To extend these results and to analyze the association of the MTNR1B- s10830963 polymorphism on fasting glucose and on prevalent type-2 diabetes risk in two elderly populations (replication cohorts); and (3) To retrospectively explore the influence of the number of full-term pregnancies on the risk of type-2 diabetes conferred by the MTNR1B polymorphism in one of the replication cohorts where this information was available.

## 2. Materials and Methods

### 2.1. Study Design and Participants

In this study, individuals (*n* = 2823) of a Spanish Mediterranean population from three defined cohorts were analyzed [61,62,63]. All the participants were recruited in the same geographical area (Valencia region). For the main aim of this work, a cross-sectional study on 1378 individuals from the general population (considered as the discovery cohort), aged between 18 and 80 years old, was undertaken, first of all in order to analyze the interaction between the MTNR1B-rs10830963 polymorphism and age in determining fasting plasma glucose. These participants were recruited in the OBENUTIC (Obesity, Nutrition & Information and Communication Technologies) study. Briefly, OBENUTIC [61] is an open case-control study carried out in the general population of the Valencia area (East Mediterranean coast of Spain). Cases were individuals with obesity (body mass index (BMI) ≥ 30 kg/m^2^) and the controls were non-obese individuals (BMI < 30 kg/m^2^) recruited from the same place and without pairing for age and sex. Cases and controls were apparently healthy individuals recruited through advertisements in shopping malls, housewives’ associations, cultural associations and other types of groups from the general population, public and private institutions, educational centers, home contacts, and primary health care centers. The exclusion criteria were being pregnant or breast-feeding, suffering from some infectious/contagious disease, invalidating physical or psychological diseases, cancer, thyroid diseases, Cushing disease, high alcohol intake, or the consumption of other drugs, as previously detailed. In this study, 1378 participants (543 men and 835 women), who had consecutively complete data available for the MTNR1B-rs10830963 polymorphism and fasting glucose concentrations, were analyzed. Of them, 53 (3.8%) were type-2 diabetics. Participants provided informed consent and study protocol and procedures were approved according to the ethical standards of the Helsinki Declaration and by the Human Research Ethics Committee of the University of Valencia, Valencia (ethical approval code H1488282121722).

Later, in order to check the possible replication of the associations of the MTNR1B-rs10830963 polymorphism with fasting glucose in a more elderly population, as well as to extend the results by testing the association between the MTNR1B-rs10830963 polymorphism and type-2 diabetes, given that prevalence was low in the general population, a replication cohort 1 was selected. This replication cohort consisted of 1001 elderly individuals (mean age 67 years, within a range of 55 to 80 years in men and 60 to 80 in women) participating in the “PREvención con DIeta MEDiterránea” (PREDIMED)-Valencia study. Details of this study have previously been published [62]. Briefly, participants were high cardiovascular risk individuals who fulfilled at least one of the following two criteria: type-2 diabetes; or having three or more cardiovascular risk factors: current smoking, hypertension (blood pressure ≥ 140/90 mmHg or treatment with antihypertensive drugs), low-density lipoprotein cholesterol (LDL-C) ≥ 160 mg/dL (or treatment with hypolipidemic drugs), high-density lipoprotein cholesterol (HDL-C) ≤ 40 mg/dL, body mass index (BMI) ≥ 25 kg/m^2^, or a family history of premature cardiovascular diseases. These participants provided written informed consent and study protocol and procedures were approved by the Human Research Ethics Committee of the University of Valencia, Valencia (ethical approval code H1422226460525). Finally, another cohort (replication cohort 2) was obtained to analyze the replication of the MTNR1B-rs10830963 SNP associations with fasting glucose and with type-2 diabetes risk in an elderly population, as well as to extend results by undertaking an additional exploratory analysis related to the influence of the number of pregnancies on the type-2 diabetes risk conferred by the polymorphism. We considered this analysis as exploratory, bearing in mind the small sample size in this cohort. This cohort consisted of participants in the PREDIMED Plus-Valencia study. The characteristics of this study have previously been published [63]. Participants were men (aged 55 to 75 years) and women (aged 60 to 75 years), with metabolic syndrome [63]. The Institutional Review Board of the Valencia University approved the study protocol (ethical approval code H1373255532771) and all participants provided written informed consent. Further, 444 individuals were included that had been recruited consecutively and for whom complete data on the MTNR1B-rs10830963 polymorphism and fasting glucose were available. Although data on gestational diabetes mellitus was not available in this elderly cohort, data on the number of full-term pregnancies in women was. In this cohort, we retrospectively explored the interaction between the number of full-term pregnancies reported by women and the MTNR1B polymorphism in the future type-2 diabetes risk (assessed as type-2 diabetes prevalence at baseline).

### 2.2. Demographic, Anthropometric, Biochemical, Clinical, and Lifestyle Variables

The assessment of the participants in the three cohorts was performed by the same researchers using similar protocols. Sociodemographic and clinical variables were obtained through the standardized questionnaires previously used and specifically designed for these cohorts [61,62,63]. Anthropometric variables and blood pressure were measured by trained staff and in accordance with the standard recommendations and the study protocol. Weight and height were directly measured with calibrated scales and a standard calibrated stadiometer [61,62,63]. Body mass index (BMI) was calculated as the weight in kilograms divided by height in square meters. Waist circumference was measured midway between the lowest rib and the iliac crest, after normal exhalation, using an anthropometric tape. Blood pressure was measured with the use of validated semiautomatic oscillometers (Omron HEM-705CP, OMRON Healthcare Europe B.V., Hoofddorp, the Netherlands), as previously reported [61,62,63]. Blood samples were collected after overnight fasting. Fasting plasma glucose, HDL-C, and triglyceride concentrations were measured as previously described [61,62,63]. LDL-C was estimated by the Friedewald equation. Fasting glucose concentrations were determined in the same clinical laboratory for all three cohorts. Clinical variables and medication used were assessed by questionnaires. Type-2 diabetes was defined as previously reported [61,62,63]. The number of full-term pregnancies (considered as a pregnancy ending in a live birth) were assessed by questionnaire in women of replication cohort 2 [63].

### 2.3. DNA Isolation and Genotyping

Genomic DNA in the three cohorts was isolated from white blood cells. DNA quality control was undertaken in the laboratory of the University of Valencia, as previously reported [61,62,63]. In the discovery cohort (OBENUTIC study), the MTNR1B-rs10830963 polymorphism was determined using an ABI Prism 7900HT Sequence Detection System (Applied Biosystems by Life Technologies, Thermo Fisher Scientific Inc., Waltham, MA, USA) and the corresponding fluorescent allelic discrimination TaqMan assays by standard procedures and quality controls in subjects with DNA available. For participants in replication cohort 1 (PREDIMED-Valencia study), the MTNR1B-rs10830963 polymorphism was determined using the Infinium OmniExpress-24 BeadChip genotyping array (v1.0 and v1.1) (Illumina Inc., San Diego, CA, USA), according to the manufacturer’s protocol, with appropriate quality standards. Allele detection and genotype calling were performed in the GenomeStudio genotyping module (Illumina Inc., San Diego, CA, USA), as previously reported [62]. The corresponding genotype data for the MTNR1B-rs10830963 polymorphism were extracted by a Phyton script that was created [62]. For participants in replication cohort 2 (PREDIMED Plus-Valencia study), the MTNR1B-rs10830963 polymorphism was determined using the Infinium OmniExpress-24 v1.2 BeadChip genotyping array (Illumina Inc., San Diego, CA, USA) at the University of Valencia, as previously reported [63]. The polymorphism was in Hardy–Weinberg equilibrium in all the three cohorts (*p* = 0.091, *p* = 0.900, and *p* = 0.889, respectively).

### 2.4. Statistical Analysis

Chi-square tests were used to compare proportions and Student’s t and ANOVA tests were applied to compare continuous variables. Crude and multivariable-adjusted models were fitted for each cohort. The MTNR1B-rs10830963 polymorphism was considered codominant (three genotypes) as well as additive (alleles 0, 1, and 2 for the minor risk allele “G”), depending on the genetic model analyzed. For the cross-sectional analysis in the discovery cohort, general linear models were used. Firstly, an unadjusted model was used to test the association between the MTNR1B-rs10830963 polymorphism and fasting glucose concentrations. The crude model was then sequentially adjusted for other potential confounders as follows: adjusted for age, sex, and type-2 diabetes and adjusted for obesity. Additional adjustment for other variables (smoking, total cholesterol, triglycerides, blood pressure, or diabetes medication) for specific analyses were carried out. When indicated, analyses were stratified for non-diabetic and type-2 diabetic subjects. The estimates were made for both the whole population (men and women jointly) and were stratified by sex to apply the sex/gender perspective to our analyses [64]. To test the interaction between the polymorphism and age in determining fasting glucose, a linear hierarchical model was used in which the main effects and the interaction term were included. The age variable was used both continuously and dichotomously (depending on the age mean) in separated models. To present the effects of the interaction using age as a continuous variable, the predicted values were computed for each individual from the adjusted regression model and these values were plotted against age in years by the MTNR1B-rs10830963 genotype, as previously described [65]. The models were also stratified by type-2 diabetes when indicated. For the study on the association between the polymorphism (additive model) and the prevalence of type-2 diabetes, logistic regression models were used. Models were both crude and sequentially adjusted. The odds ratio (OR) and the corresponding 95% confidence interval (CI) were estimated. Finally, in the women of replication cohort 2, we analyzed the interaction between the MTNR1B-rs10830963 polymorphism and the number of full-term pregnancies in determining type-2 diabetes. In this specific analysis, the “exposure” was the number of pregnancies retrospectively reported by women. The MTNR1B-rs10830963 genotype was considered the genetic risk variable. In a logistic regression model, we tested the interaction term between the number of pregnancies and the genotype. In this model, the main effects were also included and the model was additionally adjusted for covariates, as indicated in the text. The outcome variable was the current prevalence of type-2 diabetes at baseline obtained in this cohort. No information on gestational diabetes was available. The SPSS Statistics for Windows Ver. 26 (IBM Corp., Armonk, NY, USA) was used for statistical analyses. All reported probability tests were two sided. Differences were considered significant at *p* <0.05.

## 3. Results

### 3.1. Association between the MTNR1B Polymorphism and Fasting Plasma Glucose in Mediterranean Subjects Aged 18 to 80 Years (Discovery Cohort)

#### 3.1.1. Associations in the Whole Population

First of all, the association of the MTNR1B-rs10830963 polymorphism with fasting glucose was analyzed in 1378 subjects from the general population (OBENUTIC study). This cohort was considered the discovery cohort. Table 1 shows the demographic, anthropometric, biochemical, clinical, and genetic characteristics of the participant in this discovery cohort in the whole sample and by sex. The ages of these participants were wide ranging, from 18 to 80 years old; the mean age of this cohort was 41 years. For some age stratifications, the population mean was chosen to create two groups: the young age group (individuals under or equal to 41; *n* = 684 subjects) and the older group (above 41 years; *n* = 694 subjects). This cohort was relatively healthy, with a very low prevalence of type-2 diabetes (3.8%). The minor allele frequency (MAF) of the variant risk allele “G” was 0.31, similar to other European populations [29,30,31,32,33]. No difference was detected in the frequency of the MTNR1B-rs10830963 polymorphism between men and women (*p* = 0.409), nor were any differences detected in the frequency between age groups (*p* = 0.554) in those less than or greater than 41 years.

**Figure 1:**
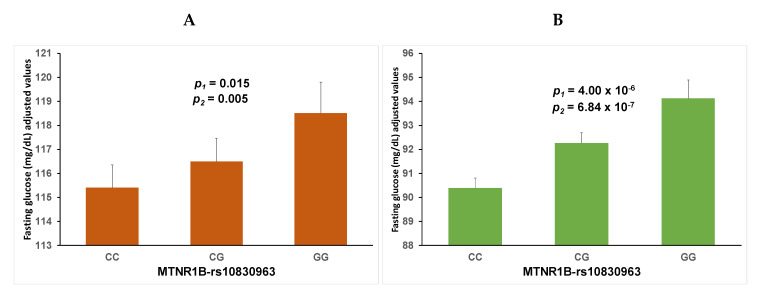
Associations between the MTNR1B-rs10830963 polymorphism and fasting glucose concentrations in: (**A**): whole population in the discovery cohort (OBENUTIC study) (*n* = 1378); (**B**): non-diabetic subjects (*n* = 1325) of the discovery cohort (OBENUTIC study). Means were adjusted for sex, age (as continuous), type-2 diabetes, and obesity. Variables are represented as means and SE. MTNR1B: melatonin receptor 1B gene; CC, CG and GG are the MTNR1B-rs10830963 genotypes. The *p*-values were obtained for the MTNR1B-rs10830963 polymorphism in the adjusted multivariable linear regression models, using two genetic models: *p*_1_ as co-dominant model; *p*_2_ as additive model.

When considering the whole population (all the age groups, diabetics, and non-diabetics; *n* = 1378 in the discovery cohort), a statistically significant association was found between the MTNR1B-rs10830963 polymorphism (codominant model) and fasting glucose (*p* = 0.009) in the unadjusted model. This polymorphism main effect remained statistically significant in the model adjusted for sex, age (as continuous), type-2 diabetes, and obesity (*p* = 0.015). Figure 1, panel A shows the adjusted means of fasting glucose by MTNR1B-rs10830963 genotypes. Also, the *p*-value for the polymorphism in the additive genetic model, adjusted for the above indicated covariates, was calculated (*p* = 0.005). The homozygous GG were those that had higher fasting glucose in comparison with the CC homozygous and in an intermediate position were heterozygous CG.

To minimize the possibility that the fasting glucose association might reflect the inclusion within the cross-sectional study samples of subjects with known type-2 diabetes, stratified analyses of the associations in non-diabetic subjects (*n* = 1325) in the discovery cohort were carried out. When only considering non-diabetic subjects in this cohort (OBENUTIC study), the association of the MTNR1B-rs10830963 polymorphism in the codominant (*p* = 4 × 10^−6^) as well as in the additive genetic model (*p* = 6.84 × 10^−7^) with fasting glucose concentrations in the model adjusted for sex, age, and obesity remained highly significant (Figure 1, panel B). In this additive model, the estimated increase in fasting glucose associated with the G-allele was B: 1.86 ± 0.37 mg/dL (*p* = 6.84 × 10^−7^). In the model for the whole population (including non-diabetic and type-2 diabetic subjects), the regression coefficient for age was B = 0.43 ± 0.03 mg/dL; *p* < 0.001, showing an increase of fasting glucose with increasing age. In non-diabetic subjects, the regression coefficient for age was B = 0.31 ± 0.02 mg/dL; *p* < 0.001. Further adjustment of this model by tobacco smoking (23.5% smokers) did not change the statistical association (*p* = 4.15 × 10^−7^). Moreover, after additional adjustment of this model for blood pressure, total cholesterol, and triglycerides, the association between the polymorphism and fasting glucose remained statistically significant (*p* = 7.0 × 10^−6^). Finally, on studying the association stratified per sex, in both non-diabetic men and women, the G allele was significantly associated with higher fasting glucose in men (*p* = 4.7 × 10^−5^) and in women (*p* = 0.002).

#### 3.1.2. Modulations by Age

The significant association of the MTNR1B-rs10830963 polymorphism with fasting glucose was initially expected, given that it had previously been reported in many studies [26,27,28,29,33,34,35,36,37,38,39,40]. However, we hypothesized that the association would be heterogeneous depending on age and not analyzing heterogeneity per age could lead to biased results when applying the conclusions to precision medicine. Therefore, the next step was to study the interaction between the MTNR1B-rs10830963 polymorphism and age (initially as a continuous variable) in determining fasting plasma glucose concentrations in the discovery cohort.

First, the statistical significance of that interaction for the whole population (*n* = 1378 subjects) was studied, including type-2 diabetic subjects and non-diabetics. In a model adjusted for sex, age (as continuous), type-2 diabetes, and obesity, a statistically significant interaction was obtained between age and the MTNR1B-rs10830963 polymorphism (*p* = 0.001). Even after additional adjustment for diabetes medication, this gene-age interaction remained statistically significant (*p* = 0.002).

**Figure 2:**
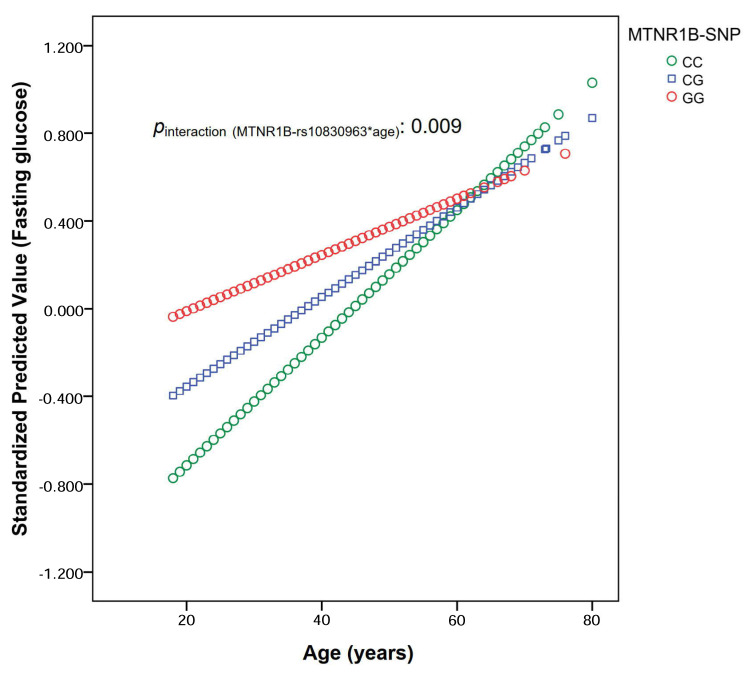
Interaction between the MTNR1B-rs10830963 polymorphism and age (as a continuous variable) in determining fasting glucose concentrations in non-diabetic subjects from the discovery cohort (OBENUTIC study) (*n* = 1325). MTNR1B: melatonin receptor 1B gene; CC, CG and GG are the MTNR1B-rs10830963 genotypes. Results obtained in a multivariable linear regression model. Model and p-value for the interaction term were adjusted for sex, age (as continuous), and obesity. *p*-interaction = 0.009 in the hierarchical multivariate adjusted model. This continuous interaction was depicted by computing the predicted fasting glucose values (standardized) for each individual from the multivariate adjusted regression model and plotting them against chronological age (in years) by MTNR1B-rs10830963 genotypes.

In the same way, as mentioned above, to obtain a better estimation of the effects on fasting glucose in non-diabetics, the gene-age interaction analysis was computed only in non-diabetic subjects (*n* = 1325) of the discovery cohort. Likewise, a statistically significant gene-age interaction was obtained both in the crude model (*p* = 0.008) and in those adjusted for age and sex (*p* = 0.012) and subsequently, for obesity (*p* = 0.009). Figure 2 shows the effect of the interaction between the MTNR1B-rs10830963 polymorphism and age (as a continuous variable) on fasting glucose concentrations in the non-diabetic participants in the discovery cohort for the model adjusted for age, sex, and obesity. In agreement with the estimated model, the differences of fasting glucose depending on the MTNR1B-rs10830963 genotype are of greater magnitude at young ages, whereas as the population ages, the effects of the genotype become smaller.

To better characterize this interaction per age, the dichotomous age variable based on the population mean (41 years) was analyzed. Table 2 presents the gene-age group interaction effects by stratifying the MTNR1B-rs10830963 polymorphism estimation on fasting glucose in the young age group (under 41 years) and in the older age group (over 41 years). Results are presented for the whole population in the discovery cohort (*n* = 1378) and in non-diabetic participants (*n* = 1325). The polymorphism was analyzed both as codominant (three genotypes) and as additive (allele-G effect). The corresponding means by genotype or the regression coefficients (depending on the model), the SE, and the statistical significance for each age-group are indicated. In addition, the p-values for the corresponding interaction terms for each statistical model are shown. All the p-values for the interaction terms between the MTNR1B-rs10830963 polymorphism and the two age groups are statistically significant, indicating a robust consistency for this interaction. Each statistical model in Table 2 provides information regarding the genotype or the G-allele effects for comparative analyses. Overall, on analyzing the effect sizes of each stratum, we observed that in the young age group, the effect size of the polymorphism on fasting glucose concentrations was very high and statistically significant (*p* < 1 × 10^−7^ for all). In contrast, in the older group, genetic effect size of the MTNR1B-rs10830963 on fasting glucose was slight and did not reach statistical significance (*p* > 0.05 for all). For example, for the whole population (*n* = 1378) of the discovery cohort, in the fully adjusted model (p_3_) and considering the polymorphism as additive, we found a statistically significant interaction term between the polymorphism and age (*p* = 0.001). In subjects aged ≤ 41 years, the regression coefficient for the multivariate-adjusted model was B = 3.00 ± 0.47 mg/dL, indicating a strong effect per G-allele increasing in fasting glucose in this age group (*p* = 4.61 × 10^−10^). However, the same model in subjects aged over 41 years revealed a non-statistically significant effect (B = −0.2 ± 0.9; *p* = 0.805) of the MTNR1B-rs10830963 polymorphisms on fasting glucose concentrations in this group.

Furthermore, homogeneity per sex was analyzed in the discovery cohort (*n* = 1378). We observed that in the stratified analysis by sex, the *p*-values for the interaction term between the MTNR1B-rs10830963 polymorphism (as additive) and age (dichotomous in 41 years) determining fasting glucose concentrations in the fully adjusted model including age, type-2 diabetes, and obesity were: *p* = 0.021 for men and *p* = 0.032 for women. Thus, the age-interaction effects for men and women were present in both.

### 3.2. Association between the MTNR1B Polymorphism and Fasting Plasma Glucose and Type-2 Diabetes in an Elderly Population (Replication Cohort 1)

Given that this is the first time that interaction per age in the effects of the MTNR1B-rs10830963 polymorphism on determining fasting glucose has been characterized, we wanted to check whether in another elderly population, the non-association of the polymorphism with fasting glucose could be replicated. A replication and extension cohort consisting of 1001 participants from the PREDIMED-Valencia study were analyzed. Table S1 shows the general characteristics of these participants according to sex (371 men and 630 women). Mean age was 66.9 ± 6.2 years (ranging from 55–80). Prevalence of type-2 diabetes in this high cardiovascular risk population was high (46.4%). In agreement with our previously described gene-age interaction in the discovery cohort (OBENUTIC population), no significant associations were detected between the MTNR1B-rs10830963 polymorphism and fasting plasma glucose in our older replication cohort 1 (*p* > 0.05 for all models and strata). For the whole replication cohort 1 (*n* = 1001) in a model adjusted for sex, age, type-2 diabetes, and obesity, the MTNR1B-rs10830963 G-allele (additive model) presented a small effect size and was not significantly associated with fasting glucose (B: 0.29 ± 1.6 mg/dL; *p* = 0.859). No significant changes were detected after additional adjustment for smoking or diabetes medication (not shown).

Table 3 shows adjusted means of fasting glucose (adjusted for sex, age, and obesity) depending on the MTNR1B-rs10830963 genotype (codominant model) and regression coefficients for the G-allele in non-diabetic and in type-2 diabetic subjects. As expected, no significant associations for the polymorphism were detected in any strata. The regression coefficient for age in this elderly cohort was B: 0.26 ± 0.13; *p* = 0.050 in non-diabetic subjects. No significant association with age was found in diabetic subjects (*p* = 0.401).

Given that the prevalence of type-2 diabetes in this replication cohort 1 is high (46.4%) compared with the discovery cohort, we analyzed the association between the MTNR1B-rs10830963 and type-2 diabetes prevalence (Table 4).

GG genotype was more prevalent in type-2 diabetic subjects than in non-diabetics. Likewise, in an additive model, the G-allele was significantly associated with type-2 diabetes risk, even after adjustment for sex, age, and obesity: OR: 1.22; 95%CI: 1.01–1.48; *p* = 0.048. Interestingly, this result shows the effect of the MTNR1B-rs10830963 polymorphism on prevalent type-2 diabetes, even in an elderly population. On analyzing homogeneity by sex, the results may suggest a relatively higher effect in women (OR: 1.24, 95%CI: 0.97–1.60; *p* = 0.092) in comparison to men (OR: 1.21; 95%CI: 0.88–1.67; *p* = 0.244) in the model adjusted for age and obesity. However, the sample size is small and no statistical differences between sexes were reached.

### 3.3. Association between the MTNR1B Polymorphism, Fasting Plasma Glucose, and Type-2 Diabetes in Another Elderly Population (Replication Cohort 2). Exploratory Analysis of the Influence of Parity in the Effects of the Polymorphism on Type-2 Diabetes

Table S2 shows the general characteristics of replication cohort 2 according to sex. Mean age was 65.2 ± 4.8 years. The prevalence of type-2 diabetes was 39%. Consistent with our previous findings in the discovery cohort as well as in replication cohort 1, in this elderly population, the MTNR1B-rs10830963 polymorphism was not significantly associated with fasting glucose concentrations. For the whole replication cohort 2, we obtained a p-value of 0.326 in the codominant model adjusted for sex, age, type-2 diabetes, and obesity. In the additive model with the same adjustments, the G-allele was not associated with fasting glucose (B: −2.04 ± 1.66 mg/dL; *p* = 0.219). Likewise, no associations were detected when we analyzed non-diabetic participants from this cohort with the same model (B: 0.22 ± 1.23 mg/dL; *p* = 0.857).

Table 5 shows adjusted means and regression coefficients for the association between the MTNR1B-rs10830963 polymorphism and fasting glucose in the replication cohort 2 stratified by type-2 diabetes status. The results were in agreement with the gene-age interaction found in our discovery cohort.

Regarding associations with type-2 diabetes risk, in replication cohort 2, the sample size was smaller than in replication cohort 1. Therefore, the effects have to be greater to be detected as statistically significant. The MTNR1B-rs10830963 polymorphism did not reach statistical significance for the whole population (OR:1.12, 95%CI: 0.85–1.49; *p* = 0.434) in an additive model adjusted for sex, age, and obesity. We explored whether the number of full-term pregnancies retrospectively reported by women interacted with the MTNR1B polymorphism and the later risk of type-2 diabetes. The number of pregnancies ranged from 0 to 7, with a distribution as follows: none (5.6%), one (10.8%), two (46.0%), three (23.6%), and four or more (14%). In a model adjusted for age and obesity, we detected a statistically significant interaction term between the MTNR1B-rs10830963 polymorphism (additive model) and the number of full-term pregnancies (as continuous), being the estimation for this interaction term: OR: 1.48, 95%CI: 1.01–2.16; *p* = 0.042, indicating that a higher number of pregnancies in carriers of the G-allele significantly increased the later risk of type-2 diabetes. Considering that these women’s mean age is 67 years, at least 25–35 years had passed by since the end of their fertile age.

## 4. Discussion

In this study, carried out on individuals of a wide age range (18 to 80) from three Mediterranean cohorts, we have been able to demonstrate that the effects of the MTNR1B-rs10830963 C>G polymorphism [33] on fasting plasma glucose concentrations are heterogeneous and greatly depend on the age of the population studied. The MTNR1B gene encodes a high affinity form of a receptor for melatonin, the primary hormone secreted by the pineal gland, under the control of the suprachiasmatic nucleus and consequently has a profound circadian rhythm [45,46,47,48,66]. Thus, plasma melatonin concentrations are high during night-time and decrease during daylight [67]. It has been suggested that melatonin could be involved in the circadian regulation of insulin concentrations through several mechanisms, including a drop of insulin levels during the night [47,48,49,68]. Initial functional studies reported that the MTNR1B was expressed in human islets and in β-cells and that expression of the MTNR1B gene was increased in G-allele carriers of the rs10830963 C>G polymorphism [42]. However, age-related effects on gene-expression were reported in this study [42]. Likewise, this variant has been associated with impaired insulin secretion and reduced acute insulin response to glucose [42]. Other studies have reported that melatonin impairs glucose tolerance in humans, especially in G-allele carriers [53]. However, currently, controversy still exists regarding the potential mechanisms involved, as well as on the beneficial or unfavorable effects of the endogenous and/or exogenous melatonin in humans [50,51,52,53,54,55,56,57,58]. Some of these controversial results may be partially explained by taking into account the existence of heterogeneous effects of the MTNR1B polymorphism by age. Therefore, depending on the age of the individuals studied, the results from different studies may differ, emphasizing the relevance of the context and leading to a more personalized conclusion and potential recommendations. An age-related chronobiological hypothesis was suggested by Hardeland [69] to explain the contradictory results on the favorable or unfavorable effects of the melatonin on glucose metabolism in humans. According to this researcher, whether melatonin may be harmful in type-2 diabetes of humans, despite its antidiabetic results in rodents [51,52,53,54,55,58], remains to be elucidated and may partly depend on age. The reductions in melatonin secretion associated with aging as well the deteriorating circadian system and the proinflammatory changes may play a major role [70].

Despite the relevance of age, its impact on the genetic effects has been largely unexplored. For the vast majority of genes involved in glucose metabolism or chronobiology, we do not know whether the magnitude of the genetic effects is constant or whether it varies with age. In an initial Genome-wide association study (GWAS) meta-analysis on more than 35,000 individuals of European descent, a highly significant association between the rs10830963 C>G polymorphism and fasting plasma glucose concentrations was reported [33]. Overall, each G allele was associated with an increase of 0.07 mmol/L (1.26 mg/dL) in fasting glucose concentrations. Subsequent studies [20,25,26,27,28,29] also reported statistically significant associations between the MTNR1B-rs10830963 polymorphism and fasting glucose concentrations, but the formal analysis of a potential gene-age interaction was ignored.

We tested the age-MTNR1B-rs10830963 polymorphism interaction in our discovery cohort and found a strong interaction effect both on considering age as a continuous variable (we detected a step-wise decrease in the genetic effect size at increasing age) and on considering age as a categorical variable (two groups based on the population mean). Therefore, we were able to confirm that the effect size of the MTNR1B-rs10830963 polymorphism on fasting glucose is greater in young people and diminishes in the older population to the point of not detecting statistically significant effects in the older groups. As far as we know, no previous study has specifically reported a statistically significant interaction between the MTNR1B-rs10830963 polymorphism and age in determining fasting glucose concentrations (mainly because it has not been formally studied). However, in some studies and meta-analyses, the existence of statistically significant heterogeneity in genetic effect sizes of the MTNR1B-rs10830963 polymorphism on fasting glucose concentrations has been reported [33,41]. That heterogeneity has mostly been attributed to ethnicity (greater effect on Europeans than on Asians) [41], with other sources of heterogeneity being less conclusive [41,53]. Although no formal gene-age interaction in the effects of the MTNR1B-rs10830963 polymorphism on fasting glucose concentrations has been explicitly reported, if we look at the mean age of participants included in the published studies, we can see that several of the initial studies were undertaken with a younger population [21,23,26]. Outstanding among these studies for its pioneering character is that undertaken by Staiger et al. [21] on 1578 individuals from Southern Germany, with a mean age of 40, in which a statistically significant association of the MTNR1B-rs10830963 polymorphism with increased fasting glucose and reduced oral glucose tolerance test (OGTT) was described. Likewise, Kelliny et al. [23] found an association between the MTNR1B-rs10830963 polymorphism and fasting glucose in healthy children in the European Youth Heart Study [23]. Barker et al. [26] conducted a genetic meta-analysis on glucose levels of over 6000 children, obtaining statistically significant results. In the pioneering meta-analysis of Prokopenko [33], including 36,610 individuals of European descent from 10 cohorts, on comparing the effects of the G allele in the different cohort, some heterogeneity per age can be observed. Thus, in the NFBC1966 study (Northern Finland Birth Cohort 1966), where the mean age of the population studied was 31 years, the effect of each G allele on fasting glucose was 0.079+/−0.012 mmol/L (1.42 mg+/-0.22 mg/dL). This contrasts with a lesser effect of the G allele in the Framingham cohort (0.050+/−0.012 mmol/L; 0.99 +/−0.22 mg/dL), where the mean age of the participants (64 years) was greater than in the NFBC1966 study. Although other factors related with the specific population characteristics may contribute to the differences, age may be a relevant one. Later, Holzapfel et al. [71], who analyzed the association of that polymorphism in children and adolescents, found that each G allele was associated with an increase of 0.205 mmol/L (3.69 mg/dL) in fasting glucose (*p* < 0.0001), so they concluded that “the effect sizes in children and adolescents seem to be stronger than in adults.” This conclusion supports our results regarding the interaction according to age and our observation that the effects are greater in a younger population than in an elderly population.

Overall, this gene-age interaction has been masked in large cohorts, including individuals with a wide age range, despite the mean age of those cohorts being elderly, due to the fact that the mean main effect of the polymorphism is relatively high and statistically significant associations of the polymorphism with fasting glucose have been found [33]. This does not contradict the gene-age interaction observed by our study, given that as we have shown in our discovery cohort, despite the strong gene interaction observed with age in the corresponding formal analysis (Figure 2, Table 4), if we do not formally examine as our main aim the analysis of the age-gene interaction between the MTNR1B-rs10830963 polymorphism and age in determining fasting glucose, the global association between the polymorphism and fasting glucose was statistically significant in the whole population (see Figure 1). Thus, results of our study may be useful for re-analyzing already published associations of the MTNR1B-rs10830963 polymorphism in other cohorts [19,21,27,29,33,41,42] and to better characterize their heterogeneity per age and population. This could be useful for obtaining more accurate information to apply to future precision medicine or precision nutrition [1,2,3]. Another advantage of our study is that it also provides an analysis in two replication cohorts. These Mediterranean cohorts both consist of an elderly population (mean age 66–67 years), in which we can replicate the non-association of the polymorphism with fasting glucose in older subjects, and also, in these cohorts, we can extend the findings by including the analysis of the associations between the polymorphism and type-2 diabetes. In both cohorts, we have confirmed the no significant associations between the MTNR1B-rs10830963 polymorphism and fasting glucose concentrations, adding more evidence to the initial observation of heterogeneity per age in our discovery cohort. Despite these advantages, our replications cohorts have the limitations of not including a wide-range age as in the discovery cohort. However, the association between the MTNR1B-rs10830963 polymorphism and fasting glucose concentrations has been widely described in other populations and the novelty for replication is precisely the non-association between the polymorphism and fasting glucose in elderly subjects.

In our studies and following the recommendations of applying the gender perspective when generating scientific results [64], we examined the possible heterogeneity and homogeneity of the results in men and in women. We observed similar results by undertaking the analysis of the interaction of the MTNR1B-rs10830963 polymorphism with age in determining fasting glucose concentrations. Both in men and in women, the effects were more significant in the younger group. Glucose homeostasis in healthy individuals is closely controlled through a complex pathway of regulatory mechanisms. Disruption of normal glucose homeostasis and large elevations of fasting glucose are hallmarks of type-2 diabetes. Another important phenotype related to the MTNR1B-rs10830963 polymorphism is the association or not with type-2 diabetes. In general, most studies have concluded that there is an association between the G allele and a greater risk of type-2 diabetes [29,31,33], even though there is considerable heterogeneity depending on ethnicity, etc. [41]. Thus, in a meta-analysis of 23 studies involving 172,963 subjects, the MTNR1B- rs10830963 polymorphism was associated with an overall random effect per G-allele OR of 1.05 (95% CI: 1.02–1.08). The effects seem higher in European populations and in the meta-analysis by Prokopenko et al. [33], the same allele was associated with an increased risk of type-2 diabetes (OR:1.09, 95%CI: (1.05–1.12), per G allele). In our discovery cohort, we did not find any association of the polymorphism with type-2 diabetes risk due to the fact that in dealing with a general population, the prevalence of type-2 diabetes is very low and we did not have sufficient statistical power to undertake that study. In replication cohort 1 (consisting of elderly subjects with a high prevalence of type-2 diabetes), we did find a significant association of the polymorphism with type-2 diabetes. Although this is an elderly population in which no cross-sectional association was observed between the MTNR1B-rs10830963 polymorphism and fasting glucose at that moment, the increased type-2 diabetes risk may be due to this polymorphism producing the harmful effect on fasting glucose at a young age (not detectable in the elderly) and that over time, that dysfunction was increasing the risk of type-2 diabetes.

On analyzing the results stratified by sex, we observed that the type-2 diabetes risk results were slightly more significant in women (borderline) than in men. That may be explained in part by considering that this polymorphism has been consistently associated with a higher risk of gestational diabetes in women [34,35,36,38,40]. In a recent meta-analysis carried out by Huang et al. [72], including more than 5000 multi-ethnic gestational diabetes cases and 5000 controls, they confirmed the significant association between the MTNR1B-rs10830963 and gestational diabetes. The estimated OR is 1.47 (1.24–1.73) in Caucasians. Hence, the increase in fasting glucose in pregnant women carrying the G-risk allele, observed at a young age, may result in a higher risk of diabetes at an elderly age. As in replication cohort 1, we did not have data available on gestational diabetes mellitus in women nor on pregnancies and we were not able to analyze these possible associations. Thus, we extended our analyses to a third Mediterranean cohort (replication cohort 2), where retrospective data on the number of full-term pregnancies were available.

It is well established that women with gestational diabetes are at increased risk for future type-2 diabetes [73]. Moreover, it has been reported that women with gestational diabetes in their first pregnancy had a 40% risk of gestational diabetes in their second pregnancy. However, the factors that determine the higher risk of type-2 diabetes are not well known and more research on genetic factors is needed [74]. The risk of type-2 diabetes can increase even more in women who put on considerable weight during pregnancy or who have multiple pregnancies, among other factors [74]. Given that the sample size of our replication cohort 2 is relatively small, we undertook an exploratory analysis to study whether a greater number of pregnancies in women interacted with the MTNR1B-rs10830963 polymorphism, exacerbating the risk of future type-2 diabetes. The number of pregnancies in this population ranged from 0 to 7 and we found a statistically significant interaction with the MTNR1B-rs10830963 polymorphism in determining type-2 diabetes, in such a way that a greater number of pregnancies increased the risk of type-2 diabetes in allele G carrying women (additive model). Although the results of this study are only exploratory and the sample size is small and requires replication with other populations, they illustrate the need to delve further into differences per sex, as well as differences per age when it comes to better understanding the effects of genetic polymorphisms before investigating gene-diet interactions for applications in precision nutrition studies. In Spain, the number of children that women have has decreased [75]. However, during the fertile age of women in these elderly cohorts, the mean number of children per women is relatively high. These results reveal additional heterogeneity when taking the interpretation of the genetic studies among populations into account. In most large cohorts, it is not usual to gather information on reproductive variables in women and without this information, important factors may be omitted that boost genetic susceptibility and we may arrive at partial conclusions. Although our study results in the Mediterranean population might not be generalized to other populations, future studies, taking into account pregnancy and other variables (sleep, etc.) related to this polymorphism [76,77] in different populations are warranted.

## 5. Conclusions

In conclusion, we have a found a statistically significant gene-age interaction between the MTNR1B-rs10830963 polymorphism and chronological age in determining fasting glucose concentrations. This heterogeneity in the association of the MTNR1B-rs10830963 with fasting glucose concentrations illustrates the importance of better understanding of the presence or absence of interactions with age on genetic markers before undertaking specific studies of gene-diet interactions. Likewise, our results suggest that on analyzing the association between the MTNR1B-rs10830963 polymorphism and type-2 diabetes risk, sex-specific variables related to pregnancy in women should be considered.

## Figures and Tables

**Table 1:** Demographic, clinical, and genetic characteristics of the participants in the discovery cohort (OBENUTIC study).

	Total (*n* = 1378)	Men (*n* = 543)	Women (*n* = 835)	*p*
Age (years)	41.3 ± 14.0	40.3 ± 13.7	42.0 ± 14.2	0.030
Weight (Kg)	73.6 ± 16.5	83.5 ± 15.9	67.1 ± 13.5	<0.001
BMI (Kg/m^2^)	26.3 ± 5.2	27.2 ± 4.9	25.7 ± 5.2	<0.001
Waist circumference (cm)	88.7 ± 14.8	95.7 ± 13.7	84.1 ± 13.6	<0.001
SBP (mm Hg)	124.5 ± 17.3	130.9 ± 16.0	120.2 ± 16.8	<0.001
DBP (mm Hg)	77.7 ± 10.3	80.3 ± 10.8	76.1 ± 9.5	<0.001
Total cholesterol (mg/dL)	204.5 ± 39.7	200.0 ± 39.5	207.5 ± 9.6	0.001
LDL-C (mg/dL)	130.9 ± 33.2	131.5 ± 33.7	130.7 ± 32.8	0.681
HDL-C (mg/dL)	59.9 ± 14.3	52.4 ± 11.3	64.8 ± 13.9	<0.001
Triglycerides (mg/dL)	103.3 ± 58.3	117.7 ± 69.9	93.9 ± 47.1	<0.001
Fasting glucose (mg/dL)	92.1 ± 1 6.9	94.0 ± 17.9	90.8 ± 16.2	0.001
Type-2 diabetes: *n*, %	53 (3.8)	23 (4.2)	30 (3.6)	0.544
Obesity: *n*, %	301 (21.8)	134 (24.7)	167 (20.0)	0.040
MTNR1B-rs10830963: *n*, %				0.409
CC	665 (48.3)	270 (49.7)	395 (47.3)	
CG	565 (41.0)	211 (38.9)	345 (41.3)	
GG	148 (10.7)	62 (11.4)	86 (10.3)	

Values are mean ± SD for continuous variables and number (%) for categorical variables. BMI: body mass index; SBP: systolic blood pressure; DBP: diastolic blood pressure; LDL-C: high-density lipoprotein cholesterol; HDL-C: low-density lipoprotein cholesterol; MTNR1B: Melatonin Receptor 1B; CC, CG and GG are the MTNR1B-rs10830963 genotypes; *p*: *p*-value for the comparisons (means or %) between men and women. Student’s *t* test was used to compare means and Chi squared tests were used to compare categories.

**Table 2:** Interaction effects between the MTNR1B-rs10830963 and age (two groups based on the population means) in determining fasting glucose in the discovery cohort (OBENUTIC study). Stratified analysis by age groups. Analysis in the whole population and in non-diabetic subjects.

	Fasting Glucose (mg/dL)						
Total Population (*n* = 1378)	≤ 41 years (*n* = 684)			> 41 years (*n* = 694)			*p_interaction_*
	CC	CG	GG	CC	CG	GG	
Codominant model	84.38 ± 0.47	86.54 ± 0.51	91.65 ± 1.45	98.06 ± 1.29	98.48 ± 1.10	96.39 ± 1.40	
	*p*_1_: 2.60 × 10^−9^			*p*_1_: 0.739			*p*_int-1_: 0.008
	*p*_2_: 2.60 × 10^−9^			*p*_2_: 0.985			*p*_int-2_: 0.006
	*p*_3_: 2.10 × 0^−9^			*p*_3_: 0.969			*p*_int-3_: 0.004
Additive model	Regression coefficient (B ± SE) per G allele			Regression coefficient (B ± SE) per G allele			
	B_1_: 3.16 ± 0.52		*p*_1_: 1.58 × 10^−9^	B_1_: −0.38 ± 1.16		*p*_1_: 0.744	*p*_int-1_: 0.005
	B_2_: 2.99 ± 0.43		*p*_2_: 5.90 × 10^−10^	B_2_: −0.12 ± 0.87		*p*_2_: 0.805	*p*_int-2_: 0.002
	B_3_: 3.00 ± 0.47		*p*_3_: 4.61 × 10^−10^	B_3_: −0.21 ± 0.86		*p*_3_: 0.870	*p*_int-3_: 0.001
**Non-diabetic subjects** **(*n* = 1325)**	**≤ 41 years (*n* = 682)**			**> 41 years (*n* = 643)**			***p_interaction_***
	**CC**	**CG**	**GG**	**CC**	**CG**	**GG**	
Codominant model	84.39 ± 0.46	86.54 ± 0.51	90.46 ± 0.96	93.64 ± 0.62	94.87 ± 0.65	95.36 ± 1.29	
	*p*_1_: 2.04 × 10^−8^			*p*_1_: 0.276			*p*_int-1_: 0.043
	*p*_2_: 2.06 × 10^−8^			*p*_2_: 0.166			*p*_int-2_: 0.049
	*p*_3_: 1.77 × 10^−8^			*p*_3_: 0.187			*p*_int-3_: 0.039
Additive model	Regression coefficient (B ± SE) per G allele			Regression coefficient (B ± SE) per G allele			
	B_1_: 2.81 ± 0.58		*p*_1_: 5.04 × 10^−9^	B_1_: 0.99 ± 0.64		*p*_1_: 0.119	*p*_int-1_: 0.021
	B_2_: 2.79 ± 0.49		*p*_2_: 4.02 × 10^−9^	B_2_: 1.12 ± 0.62		*p*_2_: 0.079	*p*_int-2_: 0.029
	B_3_: 2.79 ± 0.47		*p*_3_: 3.29 × 10^−9^	B_3_: 1.00 ± 0.61		*p*_3_: 0.109	*p*_int-3_: 0.020

Values are mean ± SE or regression coefficients (B) ± SE; MTNR1B: melatonin receptor 1B gene. CC, CG and GG are the MTNR1B-rs10830963 genotypes; G-allele is the minor risk allele. Two genetic models were computed using multivariable linear regression: A codominant model and an additive model. *p*-values were obtained for each strata and model according to three statistics: Model 1 is unadjusted; Model 2 is adjusted for sex and age; Model 3 is adjusted for sex, age, and diabetes. Finally, *p*_int-1_, *p*_int-2_, *p*_int-3_, are *p*-values for the interaction terms age-polymorphism for each model.

**Table 3:** Association between the MTNR1B-rs10830963 polymorphism and fasting glucose in the replication cohort 1 participants depending on diabetes status.

	Fasting Glucose (mg/dL)					
Total Population (*n* = 1001)	Non-Diabetic Subjects (*n* = 537)			Diabetic Subjects (*n* = 464)		
	CC	CG	GG	CC	CG	GG
Codominant model	99.77 ± 1.15	101.18 ± 1.31	99.67 ± 3.09	144.10 ± 2.92	144.39 ± 3.12	144.05 ± 6.24
	*p*_1_: 0.688			*p*_2_: 0.997		
Additive model	Regression coefficient (B ± SE) per G allele			Regression coefficient (B ± SE) per G allele		
	B_1_: 0.65 ± 1.23		*p*_1_: 0.614	B_2_: 0.08 ± 3.01		*p_2_*: 0.979

Values are adjusted means ± SE for the co-dominant model and regression coefficients (B) ± SE for the additive model. MTNR1B: melatonin receptor 1B gene; CC, CG and GG are the MTNR1B-rs10830963 genotypes; G-allele is the minor risk allele. Models, means, and *p*-values (*p*_1_ and *p*_2_) are adjusted for sex, age (as continuous), and obesity. Multivariable linear regression models were fitted.

**Table 4:** Association between the MTNR1B-rs10830963 polymorphism and type-2 diabetes risk in replication cohort 1.

Strata	Genotypes (%)					
	CC	CG	GG			
Non-diabetic subjects	52.3	40.8	6.9			
Type-2 diabetic subjects	47.8	41.6	10.6	OR and 95% CI		*p*
	*p*_trend_: 0.046			Model 1:	1.22 (1.03–1.48)	0.046
				Model 2:	1.22 (1.01–1.49)	0.046
				Model 3:	1.22 (1.03–1.48)	0.048

MTNR1B: melatonin receptor 1B gene; CC, CG and GG are the MTNR1B-rs10830963 genotypes. OR: Odds ratio; CI: Confidence interval. Genotype prevalence by diabetes status is presented in % for 537 non-diabetic subjects and 464 type-2 diabetic subjects. Logistic regression models were used. Unadjusted p-value for the trend in genotype comparisons is shown. In addition, ORs and 95%Cis for type-2 diabetes risk associated with the MTNR1B-rs10830963 polymorphism are included. An additive genetic model was considered and the OR indicates the risk per G-allele. Model 1: Unadjusted. Model 2: Adjusted for sex and age. Model 3: Adjusted for sex, age, and obesity.

**Table 5:** Association between the MTNR1B-rs10830963 polymorphism and fasting glucose in replication cohort 2 participants depending on diabetes status.

	Fasting Glucose (mg/dL)					
Total Population (*n* = 444)	Non-Diabetic Subjects (*n* = 271)			Diabetic Subjects (*n* = 173)		
	CC	CG	GG	CC	CG	GG
Codominant model	99.87 ± 1.21	100.36 ± 1.23	99.96 ± 2.68	133.35 ± 3.91	130.84 ± 4.14	121.23 ± 7.12
	*p*_1_: 0.940			*p*_1_: 0.798		
Additive model	Regression coefficient (B ± SE) per G allele			Regression coefficient (B ± SE) per G allele		
	B_1_: 0.22 ± 1.23		*p*_1_: 0.857	B_2_: 5.08 ± 3.69		*p*_2_: 0.170

Values are adjusted means ± SE for the co-dominant linear regression model and regression coefficients (B) ± SE for the additive model. MTNR1B: melatonin receptor 1B gene; CC, CG and GG are the MTNR1B-rs10830963 genotypes; G-allele is the minor risk allele. Models, means, and *p*-values (*p*_1_ and *p*_2_) are adjusted for sex, age (as continuous), and obesity.

## Supplementary Materials

The following are available online at https://www.mdpi.com/2072-6643/12/11/3323/s1. Supplemental Table S1: Demographic, clinical, lifestyle, and genetic characteristics of the participants in replication cohort 1 (PREDIMED-Valencia study) at baseline according to sex; Supplemental Table S2: Demographic, clinical, lifestyle, and genetic characteristics of the participants in replication cohort 2 (PREDIMED Plus-Valencia study) at baseline according to sex.
