# Supplementary material for: Chronological Age Interacts with the Circadian Melatonin Receptor 1B Gene Variation, Determining Fasting Glucose Concentrations in Mediterranean Populations. Additional Analyses on Type-2 Diabetes Risk
Source: Nutrients. 2020 Oct 29;12(11):3323. doi: 10.3390/nu12113323 (PMC7692445; doi:10.3390/nu12113323)
Supplement: Supplementary file 1 [file nutrients-12-03323-s001.pdf]

ONLINE SUPPORTING MATERIAL

Chronological Age Interacts with the Circadian Melatonin Receptor 1B Gene Variation Determining Fasting Glucose Concentrations in the Mediterranean Population. Additional Analyses on Type-2 Diabetes Risk

Jose V. Sorlí, Rocío Barragán, Oscar Coltell, Olga Portolés, Eva C. Pascual, Carolina Ortega-Azorín, José I. González, Ramon Estruch, Carmen Saiz, Alejandro Perez-Fidaldo, Jose M Ordovas and Dolores Corella

Supplemental Table 1..2
Supplemental Table 2..3

Supplemental Table 1. Demographic, clinical, lifestyle and genetic characteristics of the participants in the replication cohort 1 (PREDIMED-Valencia study) at baseline according to sex.

	**Total (n=1,001)**	**Men (n=371)**	**Women (n=630)**	***p***
Age (years)	66.9±6.2	66.4±6.7	67.2±5.9	0.031
Weight (Kg)	76.6±12.0	81.7±11.9	73.6±10.9	<0.001
BMI (Kg/m^2)	30.3±4.2	29.6±3.7	30.8±4.4	<0.001
Waist circumference (cm)	102.7±11.7	104.3±11.7	101.7±11.7	0.001
SBP (mm Hg)	147.0±20.2	148.3±19.8	146.3±20.5	0.130
DBP (mm Hg)	81.7±10.5	82.3±10.9	81.3±10.2	0.135
Total cholesterol (mg/dL)	208.1±39.7	200.2±37.5	212.8±40.2	<0.001
LDL-C (mg/dL)	129.2±35.6	124.8±35.1	131.7±35.7	0.003
HDL-C (mg/dL)	52.7±13.5	48.3±12.2	55.3±13.3	<0.001
Triglycerides (mg/dL)	131.5±69.6	135.2±69.5	129.4±69.7	0.165
Fasting glucose (mg/dL)	119.9±39.4	126.6±41.7	116.0±37.5	<0.001
Type-2 diabetes: n (%)	464 (46.4)	198 (53.4)	266 (42.2)	<0.001
Obesity: n (%)	502 (50.1)	161 (43.4)	341 (54.1)	<0.001
MTNR1B-rs10830963: n (%)				0.691
CC	503 (50.2)	188 (50.7)	315 (50.0)	
CG	412 (41.2)	148 (39.9)	264 (41.9)	
GG	86 (8.6)	35 (9.4)	53 (8.4)	

Values are mean±SD for continuous variables and number (%) for categorical variables. BMI: body mass index; SBP: systolic blood pressure; DBP: diastolic blood pressure; LDL-C: high-density lipoprotein cholesterol; HDL-C: low-density lipoprotein cholesterol; MTNR1B: Melatonin Receptor 1B; P: p-value for the comparisons (means or %) between men and women.

Supplemental Table 2. Demographic, clinical, lifestyle and genetic characteristics of the participants in the replication cohort 2 (PREDIMED Plus-Valencia study) at baseline according to sex.

	**Total (n=444)**	**Men (n=194)**	**Women (n=250)**	***p***
Age (years)	65.2±4.8	64.0±5.4	66.1±4.1	<0.001
Weight (Kg)	84.3±13.6	92.5±13.2	77.9±10.0	<0.001
BMI (Kg/m^2)	32.3±3.6	32.2±3.4	32.4±3.7	0.644
Waist circumference (cm)	105.9±9.9	111.0±8.7	102.1±9.1	<0.001
SBP (mm Hg)	141.8±18.5	144.1±18.2	140.0±18.5	0.020
DBP (mm Hg)	80.9±9.9	80.6±10.2	80.7±9.4	0.002
Total cholesterol (mg/dL)	195.8±37.7	187.4±38.6	202.0±35.7	<0.001
LDL-C (mg/dL)	124.5±30.8	121.1±32.3	127.2±29.5	0.038
HDL-C (mg/dL)	51.5±11.3	47.3±10.6	54.8±10.8	<0.001
Triglycerides (mg/dL)	141.0±60.5	137.6±53.8	143.6±65.3	0.303
Fasting glucose (mg/dL)	113.0±27.6	113.9±28.9	112.4±26.6	0.571
Full-term pregnancies in women: n (%)				
0			14 (5.6)	
1			27 (10.8)	
2			115 (46.0)	
≥ 3			94 (37.6)	
Type-2 diabetes: n (%)	173 (39.0)	76 (39.2)	97 (38.8)	0.930
Obesity: n (%)	307 (69.1)	137 (70.6)	170 (68.0)	0.554
MTNR1B-rs10830963: n (%)				0.572
CC	202 (45.5)	85 (43.8)	117 (46.8)	
CG	194 (43.7)	90 (46.4)	104 (41.6)	
GG	48 (10.8)	19 (9.8)	29 (11.6)	

Values are mean±SD for continuous variables and number (%) for categorical variables. BMI: body mass index; SBP: systolic blood pressure; DBP: diastolic blood pressure; LDL-C: high-density lipoprotein cholesterol; HDL-C: low-density lipoprotein cholesterol; MTNR1B: Melatonin Receptor 1B; P: p-value for the comparisons (means or %) between men and women.
